# Methods to Quantify Soft-Tissue Based Facial Growth and Treatment Outcomes in Children: A Systematic Review

**DOI:** 10.1371/journal.pone.0041898

**Published:** 2012-08-06

**Authors:** Sander Brons, Machteld E. van Beusichem, Ewald M. Bronkhorst, Jos Draaisma, Stefaan J. Bergé, Thomas J. Maal, Anne Marie Kuijpers-Jagtman

**Affiliations:** 1 Department of Orthodontics and Craniofacial Biology, Radboud University Nijmegen Medical Centre, Nijmegen, The Netherlands; 2 Department of Preventive and Curative Dentistry, Radboud University Nijmegen Medical Centre, Nijmegen, The Netherlands; 3 Department of Pediatric Medicine, Radboud University Nijmegen Medical Centre, Nijmegen, The Netherlands; 4 Department of Oral and Maxillofacial Surgery, Radboud University Nijmegen Medical Centre, Nijmegen, The Netherlands; Children’s Hospital Boston, United States of America

## Abstract

**Context:**

Technological advancements have led craniofacial researchers and clinicians into the era of three-dimensional digital imaging for quantitative evaluation of craniofacial growth and treatment outcomes.

**Objective:**

To give an overview of soft-tissue based methods for quantitative longitudinal assessment of facial dimensions in children until six years of age and to assess the reliability of these methods in studies with good methodological quality.

**Data Source:**

PubMed, EMBASE, Cochrane Library, Web of Science, Scopus and CINAHL were searched. A hand search was performed to check for additional relevant studies.

**Study Selection:**

Primary publications on facial growth and treatment outcomes in children younger than six years of age were included.

**Data Extraction:**

Independent data extraction by two observers. A quality assessment instrument was used to determine the methodological quality. Methods, used in studies with good methodological quality, were assessed for reliability expressed as the magnitude of the measurement error and the correlation coefficient between repeated measurements.

**Results:**

In total, 47 studies were included describing 4 methods: 2D x-ray cephalometry; 2D photography; anthropometry; 3D imaging techniques (surface laser scanning, stereophotogrammetry and cone beam computed tomography). In general the measurement error was below 1 mm and 1° and correlation coefficients range from 0.65 to 1.0.

**Conclusion:**

Various methods have shown to be reliable. However, at present stereophotogrammetry seems to be the best 3D method for quantitative longitudinal assessment of facial dimensions in children until six years of age due to its millisecond fast image capture, archival capabilities, high resolution and no exposure to ionizing radiation.

## Introduction

Longitudinal quantitative evaluation of facial dimensions of an individual patient could inform healthcare professionals about growth as well as treatment changes [1], [2]. Accurate quantitative evaluation of craniofacial dimensions by comparison of an individual patient to normative values can provide insight into an underlying pathologic process or create a basis for treatment planning [3], [4].

Various methods for quantitative evaluation of craniofacial dimensions have been described for a variation of purposes. The standard technique is direct anthropometry which was extensively used for the study of craniofacial dimensions in the past century [5]. These “direct” measurements are reliable, inexpensive to make and regarded as the gold standard. Limitations include its time-consuming nature, the need for patient compliance and to remain still [6]. Additionally, it is not possible to archive craniofacial surface morphology. Also used for decades are two dimensional (2D) x-ray cephalometry [7]–[9] and photography [3], [10] and even today these are the most commonly used records for dento-skeletal and facial diagnosis. The advantages of these 2D imaging techniques are rapid acquisition, archival capabilities and low cost. Limitations include measurement error due to magnification, parallax and head orientation [11] and exposure to ionizing radiation. Recent technological advancements have led craniofacial researchers and clinicians into the era of three dimensional (3D) digital imaging. Techniques like cone beam computed tomography (CBCT) [12], [13], surface laser scanning [14], [15] and stereophotogrammetry [16]–[18] became available to describe and compare 3D facial surfaces, create a diagnosis or virtual treatment planning as well as to evaluate growth and treatment outcomes. These methods allow images to be archived and avoid measurement errors that occur with 2D representations of 3D surfaces. CBCT however, is not an ideal technique for surface measurement because of poor resolution of facial contours, high cost and exposure to ionizing radiation [19]. Laser surface scanning can be reliable and accurate for identifying craniofacial surface landmarks and is relatively inexpensive. Limitations include slow image capture (up to 20 seconds) and potential damage to the eyes [20]. This is particularly difficult for children because they are less able to maintain posture for this period of time and keep their eyes closed. 3D stereophotogrammetry overcomes the limitations of surface laser scanning. It is millisecond fast and has archival capabilities for subsequent morphometric studies, a good-resolution color representation and no exposure to ionizing radiation [19], [20]. The disadvantages of stereophotogrammetry are its expense, difficulties in imaging transparent, shiny and shadowed surfaces and inability to measure bony landmarks.

Many studies address validity, accuracy and reliability of craniofacial anthropometric measurements [6], [11], [21]–[25]. Differences in types of error, ages of samples and anatomical location of analysis make it difficult to compare reliability. Therefore, the objectives of this systematic review are 1) to give an overview of soft tissue-based methods for quantitative longitudinal assessment of facial dimensions in children until six years of age, 2) to assess the methodological quality of the studies using such a method and 3) to assess reliability of these imaging methods used in studies with good methodological quality.

## Methods

### Protocol and Registration

Inclusion criteria and methods of analysis were specified in advance and documented in a protocol. PROSPERO [26] for prospective registration of systematic reviews was in development at the start of this review. A registration number is therefore not available.

### Eligibility Criteria

Eligible for inclusion were primary publications which report of: 1) soft-tissue based evaluation of head and face; 2) children before 6 years of age at the start of the study; 3) quantitative changes; 4) longitudinal studies.

Excluded were publications which report of: 1) skeletal changes; 2) fetal growth; (2) animal studies, (3) cross-sectional studies, (4) case reports, reviews and letters. No restrictions for language, publication date and publication status were imposed.

### Information Resources

Studies were identified by searching electronic databases. The search was applied to PubMed (from 1948), EMBASE Excerpta Medica (from 1980), Cochrane Library (from 1993), Web of Science (from 1945), Scopus (from 2004) and CINAHL (from 1982). The last search was run on October 1, 2011. In addition, we hand searched the reference lists of included studies for potentially eligible studies. Digital full text publications were retrieved from licensed digital publishers and paper publications were retrieved from the library. In cases where the full text publication could not be retrieved, authors were requested by e-mail to deliver the publication. Gray literature was not searched.

### Search Strategy

The search strategy was developed and databases were selected with the help of a senior librarian specialized in health sciences. Databases selected were PubMed, EMBASE Excerpta Medica, Cochrane Library, Web of Science, Scopus and CINAHL. Medical Subject Headings and free text words were used for the search strategy of PubMed (Table 1). The search strategies for the other databases are directly derived from the former. The last search was performed on October 1, 2011.

**Table 1:** Search strategy PubMed.

Search strategy PubMed
(“Face”[Mesh:noexp] OR face[TiAb] OR facial[TiAb] OR craniofacial[TiAb] OR OR OR born*
craniomaxillofacial[TiAB] OR maxillofacial[TiAb] OR dentofacial[TiAb] OR “Facies”[Mesh]
facies[TiAb] OR “Head”[Mesh:noexp] OR head[TiAb]) AND (“Growth and
Development”[Mesh:noexp] OR “Growth”[Mesh:noexp] OR “growth and development”[Sh]
growth[TiAb] OR “Anthropometry”[Mesh:noexp] OR anthropometr*[TiAb] OR
“cephalometry”[Mesh] OR cephalometr*[TiAb] OR “imaging, three-dimensional”[MeSH
Terms] OR “three-dimensional imaging”[TiAb] OR “3d imaging”[TiAb] OR
“Photogrammetry”[Mesh] OR photogrammetry[TiAb] OR “Tomography, X Ray
Computed”[Mesh] OR “Tomography, X Ray Computed”[TiAb] OR “Lasers”[Mesh:noexp] OR
laser[TiAb] OR “Magnetic Resonance Imaging”[Mesh:noexp] OR “magnetic resonance
Imaging”[TiAb] OR MRI[TiAb]) AND (infant OR infan* OR newborn OR newborn* OR new
OR baby OR baby* OR babies OR neonat* OR perinat* OR postnat* OR toddler* OR
kindergar* OR preschool* OR pre school) AND (“Cohort Studies”[Mesh] OR ((cohort[TiAb]
OR longitudinal[TiAb] OR followup[TiAb] OR follow up*[TiAb]) AND (study[TiAb] OR
studies[TiAb])))

The search strategy focused on four aspects:

1. terms to search for the population of interest (*i.e*., baby’s, infants and pre- school children). A selection of the appropriate terms from the Child search strategy was made to sort out citations not reporting on children between 0 and 6 years of age [27];
2. terms to search for growth and methods for quantitative evaluation (*i.e.*, growth, anthropometrics and imaging techniques);
3. terms to search for the anatomic region of interest (*i.e.*, face and head);
4. terms to search for the longitudinal aspect (*i.e.*, cohort and follow up studies).

### Study Selection

First, studies were independently screened on title and abstract by two reviewers (SB and MB) in a blinded standardized manner. In an additional step, disagreements between reviewers were resolved by discussion and consensus.

Second, full text assessments for eligibility were independently performed by two reviewers in a blinded standardized manner. In an additional step, disagreements were resolved by discussion and consensus.

Third, a hand search of the reference lists of the included studies was performed by the first author.

Finally, all included studies were categorized as describing facial or cranial evaluation of growth and treatment outcome. The plane connecting glabella with left and right euryon arbitrarily separates the cranium from the face. Measurement on or above this plane are called to be cranial, below this plane are called to be facial. The studies describing facial evaluation of growth and treatment are included in this review for quality assessment. Results of the selection process by two reviewers (SB and MB) were analyzed to assess interrater reliability.

### Quality Assessment

Study quality was assessed by the quality assessment instrument (QAI) for clinical trials used by Gordon et al. (Table 2) [28]. This instrument includes an assessment of study bias. A checkmark was scored when a criterion was fulfilled. Depending on study design quality assessment was performed on a maximum of 15 criteria. In case criteria were not applicable to a certain study design, less than 15 criteria were scored. Study quality is expressed as the percentage of criteria fulfilled in relation to the total number of applicable criteria.

**Table 2:** Quality assessment instrument [28].

I. Study design (7 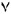 )
A. Objective–objective clearly formulated ( 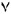 )
B. Sample size–considered adequate ( 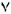 )
C. Sample size–estimated before collection of data (?)
D. Selection criteria–clearly described ( 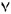 )
E. Baseline characteristics–similar baseline characteristics ( 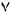 )
F. Timing–prospective ( 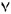 )
G. Randomization–stated ( 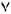 )
**II. Study measurements (3 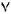 )**
H. Measurement method–appropriate to the objective ( 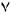 )
I. Blind measurement–blinding ( 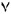 )
J. Reliability–adequate level of agreement ( 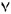 )
**III. Statistical analysis (5 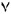 )**
K. Dropouts–dropouts included in data analysis ( 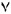 )
L. Statistical analysis–appropriate for data ( 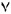 )
M. Confounders–confounders included in analysis ( 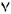 )
N. Statistical significance level–*P* value stated ( 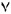 )
O. Confidence intervals provided ( 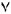 )

Maximum number of 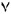
s = 15.

The score per study is calculated as a percentage by dividing the number of checkmarks by the number of applicable criteria and multiplying by 100. Studies were grouped according to similarity of the methods for measurement of facial growth or treatment outcome. A mean quality score for each group of methods was calculated. Arbitrarily, a cut-off of 60% or higher is graded as good quality. Below 60% is graded as poor quality. To assess the interrater reliability of the assessment of study quality 19 randomly selected studies were scored by two reviewers (SB and AK).

### Data Extraction

Methods, used in studies with good methodological quality, were assessed for reliability expressed as the magnitude of the measurement error and the correlation coefficient between repeated measurements.

### Statistics

Cohen’s kappa statistics were used to assess the interrater agreement for the process of study selection and for each criterion of the quality assessment instrument. According to Landis and Koch the level of interrater agreement is very good if the value of K is 0.81–1.00, good if K is 0.61–0.80, moderate if K is 0.41–0.60, fair if K is 0.21–0.40 and poor if K is <0.20 [29].

Analysis of variance (ANOVA) and non-parametric Kruskal-Wallis test were performed to test differences in mean scores between groups of methods. Fisher’s exact test was performed to test for differences between groups of methods with the use of a cut-off of 60%. SPSS version 19.0 was used as statistical software.

## Results

### Study Selection

Interexaminer kappa for screening on title and abstract was 0.76. For full text assessment of eligibility kappa was 0.69. The reliability of both steps in the process of study selection is qualified as good [29].

**Figure 1:**
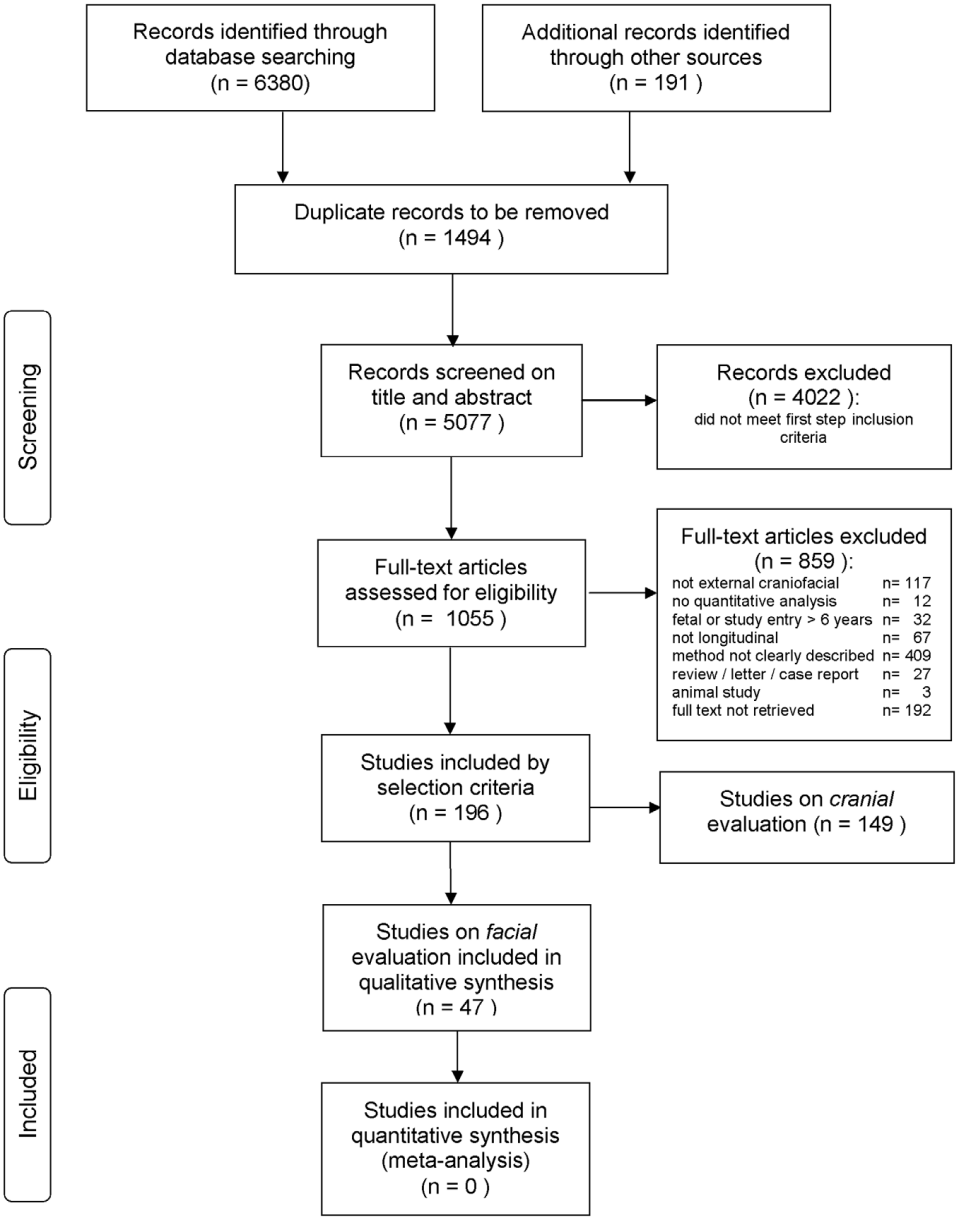
PRISMA flow diagram of study selection process.

The search of PubMed, EMBASE, Cochrane Library, Web of Science, Scopus and CINAHL provided a total of 6380 citations and the hand search provided 191 citations. After adjusting for duplicates 5077 remained for screening of title and abstract. Of these, 4022 studies were discarded because these did not meet the eligibility criteria. A total of 1055 studies remained for full text assessment of eligibility. Of these, 859 studies were excluded with reasons. Of these excluded studies, 192 were discarded because the full text publication could not be retrieved. The last step in the inclusion process divided the studies into facial evaluation (n = 47) and studies on cranial evaluation (n = 149). A total of 196 studies was identified meeting the inclusion criteria; 175 studies originated from the electronic databases; the remaining 21 studies originated from the additional handsearch of the references of the included studies. Figure 1 shows the PRISMA flow diagram and figure S1 shows the PRISMA checklist [30]. This study is restricted to studies on facial evaluation of growth and treatment outcome in children.

Of the 47 included studies, 24 studies used 2D x-ray cephalometry [31]–[54], 9 studies used 2D photography [55]–[63], 7 studies used anthropometry [64]–[70] and 7 studies used 3D imaging (2 surface laser scanning [71], [72], 4 stereophotogrammetry [73]–[76] and 1 CBCT [77]).

### Study Quality Assessment

Interrater reliability for all 15 criteria of the quality assessment instrument were between 0.19 and 1 (interexaminer kappa), 11 out of 15 criteria had a kappa of 0.50 or higher. Interrater agreement on criteria E (similar baseline characteristics), I (blind measurement) and K (dropouts included in data analysis) were below 0.20.

All included studies could be categorized into one of following methods for quantitative evaluation of soft-tissue based growth or treatment changes: 2D X-ray cephalometry, 2D photography, direct and indirect anthropometry, and 3D imaging techniques (surface laser scanning, stereophotogrammetry, cone beam computed tomography). Assessment of methodological quality of all reviewed studies resulted in scores ranging from 30% to 100%. 24 studies qualified as good according to a methodological quality score equal to or above 60%. Score summaries are shown in Table 3.

**Table 3:** Methodological quality scores of studies reporting on soft tissue-based quantitative longitudinal assessment of facial dimensions in children until six years of age.

First author	Year	Design							Measure			Statistics					Score
		A	B	C	D	E	F	G	H	I	J	K	L	M	N	O	
*2D x-ray cephalometry*																	
Bishara	1998	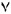	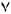	o	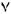	.	o	.	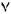	.	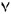	.	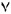	.	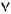	o	70%
Bishara	1997	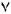	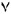	o	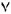	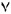	o	.	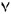	o	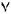	.	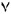	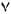	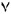	o	69%
Bishara	1985	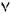	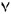	o	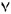	.	o	.	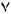	.	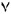	.	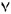	.	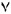	o	70%
Bishara	1985	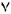	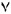	o	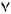	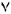	o	.	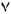	o	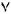	.	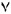	o	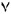	o	62%
Bishara	1984	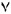	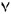	o	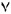	.	o	.	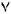	.	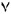	.	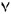	.	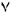	o	70%
Bongaarts	2009	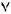	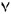	o	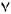	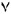	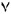	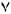	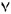	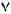	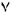	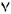	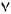	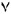	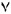	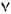	93%
Coccaro	1965	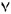	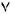	.	o	o	o	.	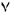	.	o	.	o	.	o	o	30%
Daskalogiannakis	2006	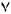	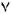	o	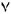	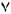	o	.	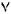	o	o	.	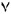	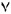	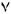	o	62%
Graber	1977	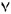	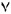	o	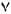	.	o	.	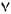	o	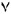	.	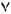	.	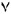	o	64%
Hanada	1975	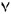	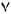	.	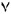	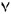	o	.	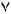	o	o	.	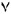	.	o	o	55%
Hermann	2004	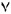	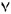	o	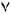	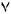	o	.	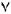	o	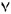	.	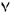	.	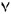	o	75%
Hermann	2003	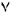	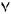	o	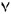	o	o	.	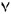	o	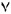	.	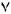	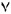	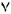	o	62%
Hermann	2003	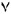	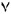	o	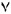	o	o	.	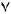	o	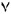	.	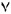	.	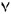	o	58%
Hermann	2002	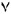	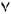	o	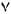	o	o	.	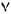	o	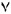	.	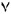	.	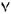	o	58%
Hermann	1999	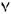	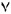	o	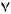	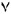	o	.	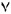	o	o	.	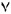	.	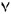	o	58%
Padwa	1999	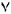	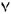	o	o	o	o	.	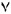	o	o	.	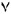	o	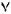	o	38%
Posen	1967	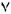	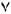	o	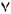	.	o	.	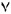	.	o	.	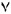	.	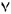	o	60%
Sadowsky	1973	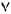	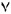	o	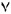	.	o	.	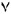	.	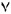	.	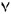	.	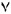	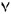	80%
Semb	1991	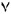	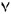	.	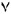	.	o	.	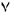	o	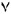	.	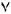	.	o	o	60%
Semb	1991	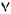	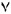	.	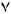	.	o	.	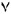	o	o	.	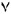	.	o	o	50%
Smahel	1995	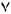	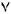	o	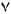	.	o	.	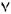	.	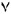	.	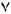	.	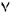	o	70%
Subtelny	1959	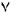	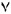	.	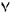	.	o	.	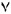	.	o	.	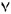	.	o	o	56%
Wen-Ching Ko	2000	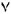	o	o	o	.	o	.	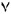	.	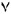	.	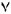	.	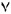	o	50%
Zettergren-Wijk	2006	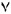	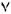	o	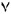	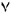	o	.	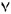	o	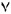	.	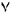	.	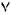	o	75%
*2D photography*																	
Altug-Atac	2008	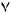	o	.	o	.	o	.	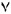	.	o	.	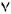	.	o	o	33%
Castelo	2010	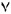	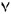	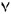	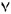	.	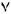	.	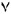	.	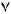	o	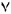	.	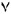	o	82%
Cruz	2008	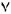	o	o	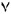	.	o	.	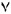	o	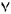	.	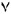	.	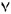	o	55%
Ko	2004	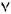	o	.	o	.	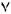	.	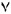	o	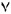	o	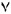	.	o	o	45%
Kohout	1998	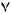	o	o	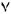	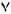	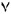	o	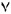	o	o	o	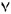	o	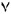	o	47%
Liou	2007	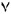	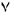	o	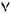	.	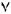	.	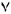	o	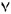	.	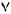	.	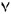	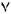	82%
Pai	2005	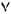	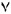	o	o	.	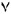	.	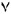	.	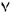	o	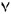	.	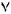	o	64%
Schüler	2007	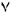	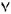	.	o	.	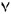	.	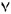	.	o	o	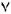	.	o	o	50%
Sultana	2000	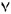	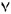	o	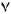	.	o	.	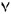	o	o	.	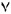	.	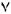	o	55%
*Direct anthropometry*																	
Bennun	1999	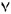	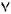	o	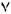	o	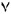	.	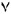	o	o	o	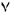	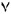	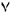	o	57%
Hansen	1997	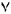	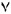	o	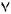	o	o	o	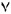	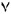	o	.	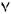	o	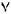	o	50%
Heimer	2008	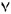	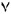	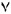	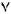	.	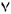	.	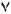	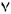	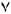	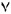	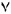	.	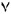	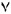	100%
Ridgway	2011	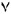	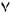	o	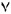	.	o	.	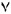	.	o	.	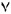	.	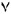	o	60%
Vander Woude	1997	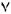	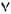	o	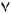	.	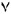	.	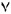	.	o	o	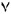	.	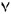	o	64%
Yang	2009	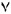	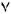	o	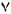	.	o	.	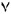	.	o	.	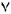	.	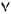	o	60%
*Indirect anthropometry*																	
Ezzat	2007	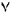	o	o	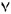	.	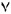	.	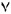	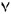	o	.	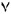	.	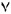	o	64%
*3D surface laser scanning*																	
Primozic	2009	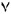	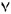	o	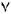	o	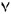	.	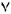	o	o	o	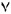	.	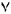	o	54%
Schwenzer	2008	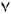	o	.	o	o	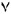	.	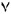	.	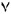	.	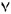	.	o	o	50%
*3D stereophotogrammetry*																	
Hoefert	2010	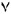	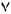	o	o	o	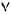	.	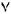	.	o	o	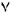	.	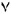	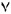	58%
Hood	2003	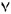	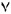	o	o	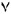	o	.	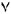	.	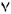	.	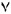	.	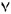	o	64%
Ras	1995	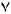	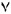	o	o	o	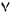	.	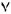	.	o	o	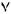	.	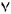	o	50%
Singh	2005	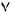	o	o	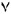	.	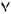	.	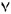	o	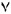	o	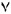	.	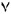	o	58%
*Conebeam computed tomography*																	
Seidenstricker	2008	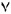	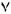	.	o	.	o	.	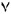	.	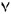	.	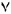	.	o	o	56%

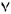
  =  Fulfilled satisfactorily the methodological criteria;

o  =  Did not fulfil the methodological criteria;

.  =  Not applicable.

Analysis of variance (*p = *0.41) and Kruskal-Wallis test (*p* = 0.15) showed no statistical significant difference for quality expressed as a percentage between groups of methods. Also Fisher’s exact test (*p* = 0.07) showed no statistical significant difference in the amount of studies with good methodological quality between groups of methods.

### Reliability

Scores for reliability of methods for soft-tissue based quantitative longitudinal assessment are shown in Table 4.

**Table 4:** Reliability of methods for soft tissue-based quantitative longitudinal assessment of facial dimensions in children until six years of age in studies with good methodological quality.

First author	Year	Measurement error	Correlation coefficient
2D x-ray cephalometry			
Bishara	1998	0.5 mm/0.5°	.
Bishara	1997	0.5 mm/0.5°	.
Bishara	1985	0.5 mm/0.5°	.
Bishara	1985	0.2 mm/0.5°	.
Bishara	1984	0.2 mm/0.5°	.
Bongaarts	2009	.	0.655–0.989
Daskalogiannakis	2006	.	.
Graber	1977	ns	.
Hermann	2004	0.27–1.94 mm/0.36–2.97°	.
Hermann	2003	0.27–1.94 mm/0.36–2.97°	.
Posen	1967	.	.
Sadowsky	1973	.	.
Semb	1991	ns	.
Smahel	1995	.	0.95–0.97
Zettergren-Wijk	2006	0.86 mm/0.80°	.
2D photography			
Castelo	2010	0.01 (ratio)	.
Liou	2007	.	0.9956
Pai	2005	ns	.
Direct anthropometry			
Heimer	2008	.	0.96–1,0
Ridgway	2011	.	.
Vander Woude	1997	.	.
Yang	2009	.	.
Indirect anthropometry			
Ezzat	2007	.	.
3D stereophotogrammetry			
Hood	2003	0.5 mm	.

.  =  not reported.

ns  =  not significant.

All good quality studies using 2D x-ray cephalometry report a measurement error below 1 mm and 1° except for the studies of Hermann et al. [41], [42] where the range is up to 2 mm for linear and 3° for angular measurements. Correlation coefficients between repeated measurements range from 0.665 to 0.989 and are qualified as good to very good. Two studies report on reliability as “no significant” error and three studies do not report on reliability at all.

Studies with good methodological quality using 2D photography report a measurement error of 0.01 in case of ratios [56], “no significant” error [61] and a correlation coefficient of 0.9956 [60] which can be qualified as very good.

No studies with good methodological quality using direct or indirect anthropometry in children below 6 years of age report on measurement error. One study reports a correlation coefficient of 0.96 to 1.0 which can be qualified as very good [66].

One study with good methodological quality using 3D stereophotogrammetry reports a measurement error of 0.5 mm [74].

There are no good quality studies using 3D surface laser scanning or CBCT in children below 6 years of age.

## Discussion

### Summary of Evidence

The objectives of this systematic review were 1) to give an overview of soft tissue-based methods for quantitative longitudinal assessment of facial dimensions in children until six years of age and 2) to assess the methodological quality of the studies using such a method and 3) to assess reliability of these quantitative measurement methods used in studies with good methodological quality. 2D X-ray cephalometry is the method used most often and has demonstrated its potential to be used in studies with a good methodological quality. Also 2D photography and anthropometry are used in studies with good methodological quality. However, only one study using 3D imaging has shown its use with a good methodological quality despite its potential benefits. A possible explanation might be that researchers pioneering these relatively new methods are more focused on application of these methods than on development of the best possible study design. Future studies using 3D imaging for quantitative evaluation of facial growth and treatment outcome should focus on proper design to demonstrate its potential to be used in studies with good methodological quality in order to take advantage of their benefits.

In literature various terms to describe the measurement error exist. Some studies use accuracy to describe landmark identification error which in turn may consists of operator error, capture error and registration error [78]. More often in literature reliability is used to describe landmark identification error of a method. Reliability can be expressed by the measurement error or correlation coefficient between repeated measurements [11], [25], [79]. Reliability represents the ability of observers to make a consistent analysis. In this systematic review reliability in studies with good methodological quality is assessed and expressed by duplicate measurement errors and correlation coefficients between repeated measurements.

Reliability in included studies using 2D x-ray cephalometry report a measurement error below 1 mm and 1°. Correlation coefficients range from 0.665 to 0.989 and are qualified as good to very good. This is in concordance with the reported reliability of digital 2D x-ray cephalometry in older children (from 9.2–11.0 years) [79]. Reliability in one of the included studies using 2D photography is qualified as very good. This is in partial agreement with Farkas et al. [11] who found only 20 out of 62 measurements to be reliable in adolescents with a measurement error equal to or below 1 mm and 2°. It is key to select reliable measurements when using 2D photography. Reliability in one of the included studies using anthropometry is qualified as very good. Well-trained anthropologist are indeed able to reliably measure craniofacial dimensions, as was shown for older individuals [5]. Finally, reliability of one included study using 3D stereophotogrammetry is good with a measurement error of 0.5 mm. This is in agreement with literature with reported measurement errors in adults between 0,20 mm and below 1 mm and a correlation coefficient of 0.91 [25], [78].

When comparing the accuracy of a technique to the standard technique or the gold standard, anthropometry -direct anthopometric measurements- correlated highly with digital 3D stereophotogrammetry (mean r = 0.88) [25]. Furthermore, millisecond fast image capture, archival capabilities for subsequent morphometric studies, a good-resolution color representation and no exposure to ionizing radiation make stereophotogrammetry the best 3D method for quantitative longitudinal assessment of facial dimensions in children until six years of age.

### Limitations

Failure to identify all relevant reports for a systematic review could result in bias [80]. For this reason highly sensitive search strategies were developed with the help of a senior librarian specialized in health sciences for a combination of both narrow and broad health science databases.

The process of study selection was performed in an independent blinded standardized manner by two reviewers to prevent unjustified exclusion of eligible studies. The hand search of the reference lists of the included studies was performed by only one reviewer. Possibly eligible studies could have been missed in this stage of the selection process. However, since only approximately one out of ten studies was retrieved by the hand search this might be negligible. Furthermore, failure to retrieve full text publications of possibly eligible studies (n = 192) was inevitable even though every effort was made to contact the authors by email in cases where online access was not permitted or the journal was not available in the library. It is estimated that approximately 8 additional studies would have been eligible for inclusion in this review.

The instrument to assess methodological quality is adapted from Gordon et al. [28] and Lagravère et al. [81]. The majority of interrater disagreements arose in the assessment of applicability of criteria E, I and K to certain studies (similar baseline characteristics, blind measurement and dropouts included in data analysis respectively). This can be explained by the absence of adequate instructions of this QAI together with the presence of a wide variety of study designs. Therefore raters should test this QAI thoroughly and obtain consensus before scoring. In literature, there is not one single tool that is an obvious candidate for assessment of methodological quality of non-randomized studies [82]. Attempts to validate QAI’s like the Newcastle-Ottowa [83] scale or the Jadad scale [84] are found to produce highly arbitrary results and are unable to demonstrate significant effects on quality scores [85], [86]. There is a need for a validated quality assessment instrument preferably applicable to a wide range of study designs. Furthermore, published studies are very often incomplete, cryptic, or written in a form unsuitable for quality assessment [87]. In order to overcome this drawbacks in future review studies, it should be recommended to publish only complete, unambiguous reports.

### Conclusions

Current 3D imaging techniques have not yet demonstrated their full potential to be used for quantitative longitudinal assessment of facial dimensions in children until six years of age. So far, stereophotogrammetry has been validated and has shown to be reliable and accurate. Its fast image capture, archival capabilities for subsequent morphometric studies, good-resolution color representation and no exposure to ionizing radiation make stereophotogrammetry at present the best 3D method for quantitative longitudinal assessment of facial dimensions in children until six years of age.

## Supporting Information

Figure S1 PRISMA checklist.
(DOC)
